# Editorial: Non-invasive Brain Stimulation in Neurology and Psychiatry

**DOI:** 10.3389/fnins.2016.00574

**Published:** 2016-12-15

**Authors:** Ignacio Obeso, Antonio Oliviero, Marjan Jahanshahi

**Affiliations:** ^1^Centro Integral en Neurociencias A.C., HM Puerta del Sur, CEU-San Pablo UniversityMadrid, Spain; ^2^Centro de Investigación Biomédica en Red, Enfermedades NeurodegenerativasMadrid, Spain; ^3^FENNSI Group, Hospital Nacional de Parapléjicos, Servicio de Salud de Castilla-La ManchaToledo, Spain; ^4^Sobell Department of Motor Neuroscience and Movement Disorders, UCL Institute of NeurologyLondon, UK

**Keywords:** neuromodulation, brain stimulation, rTMS, tDCS

In recent years, greater attention has been paid to alternative treatments in neurology and psychiatry, with the main aim of restoring or “normalizing” function in aberrant brain circuits, in order to have a positive impact on the patient's quality of life. Non-invasive brain stimulation (NIBS) methods such as transcranial magnetic stimulation (TMS) or transcranial direct current stimulation (tDCS) have been increasingly used not only in research but also in clinical settings. To date, depression is the only psychiatric disorder for which TMS has been approved and used extensively as a therapeutic approach (Padberg and George, 2009; George et al., 2013). Meanwhile, application of NIBS for other brain disorders such as tinnitus, chronic pain, migraine, dementia, Parkinson's disease (PD), and dystonia are currently in development by optimizing key parameters such as the most appropriate brain target, stimulation protocols and candidate symptoms to treat. Thus, while there has been relatively wide interest in clinical applications of NIBS, yet with refinement of techniques, future improvement of protocols and the possibility of achieving more prolonged and longer-lasting beneficial effects, we believe NIBS will potentially become an approved therapeutic approach for some disorders. The current Special Issue is a compilation of literature reviews or experimental studies using TMS or tDCS as a therapeutic tool in different neurological and psychiatric disorders.

## Non-invasive brain stimulation methods as therapeutic tools

The 16 papers in the current Research Topic demonstrate the value of NIBS in the psychiatry and neurology domains and also in cognitive training.

Evidence reveals TMS (Dunlop et al.) and tDCS (Sauvaget et al.) as effective methods for reducing craving in people suffering from eating disorders. A comprehensive review of eating disorders (anorexia, bulimia, and binge eating) confirms the positive use of repetitive TMS (rTMS) to reduce relapse rates. The suggested brain target area is the dorsolateral prefrontal cortex (DLPFC), with incremental clinical success with 10 repeated stimulation sessions. The clinical changes are considered to be potentially associated with improved cognitive control or conflict processing (Dunlop et al.), both prefrontal cortex functions. Similar results have been shown with tDCS, although in fewer studies (Sauvaget et al.). These studies used clinical ratings by patients as measures of stimulation induced change, as they are considered to more accurately reflect the patient's experience and expectations, albeit that they are subject to the common biases of self-report measures, highlighting the need for inclusion of sham-controlled conditions to control for potential placebo effects. A validated method is to combine brain stimulation with imaging (Bestmann et al., 2004). In fact, imaging has proved essential in understanding the positive response to TMS in depression, as shown by a link between clinical improvement and changes in cingulate activity (Fox et al., 2012).

A succinct overview on use of NIBS for auditory hallucinations highlights the efficacy of rTMS and TDCS in reducing the frequency of hallucinations (Moseley et al.). Higher temporo-parietal junction activity (mainly left-sided) is a potential source of hallucinations (Homan et al., 2012), which identifies this as the target location for NIBS. Repeated sessions during 5 consecutive days of cathodal tDCS reduced the hallucinations and this improvement persisted for a 3-month period. In their review, the authors considered the value and efficacy of transcranial random noise stimulation (tRNS) or transcranial alternating current stimulation (tACS) as potential future treatments for hallucination. An additional meta-analysis on conversion disorder shows in 75/86 patients under rTMS treatment a marked improvement as measured by clinical scales (Schönfeldt-Lecuona et al.), which gives further support for NIBS tools in complex neuropsychiatric conditions.

In recent years, application of NIBS in the treatment of neurological patients has been gaining pace and the use of both TMS and tDCS in neurological conditions such as stroke (Corti et al., 2012), tinnitus (Fregni et al., 2006), and PD (Koch et al., 2009) has been evaluated (for review see Obeso et al.). Yet, proof-of-principle studies are needed in treating specific neurological symptoms and to date beneficial changes are limited to acute effects, with limited long-lasting effects. Following the NIBS research approach in depression, larger and well-controlled clinical trials (i.e., use of placebo condition and coils), with longer follow-up periods are urgently needed to confirm the value of stimulation protocols with enhanced durability of clinical benefits.

TMS is useful for differential diagnosis in tremor or stroke by using motor evoked potentials (Brum et al.). Moreover, a classical clinical use of TMS has been to measure cortico-spinal integrity through examining the functioning of the cortico-spinal tracts after stimulation of motor regions. This method is adequate for differential diagnosis based on central motor conduction time (the time taken from TMS pulse activation of the motor cortex and firing of spinal motor neurons). The use of TMS and diffusion tensor imaging showed in stroke patients a correlation between the speed of conduction in the cortico-spinal tract and the integrity of premotor and supplementary tracts (but not the motor area) (Potter-Baker et al.). Their results are of interest for understanding how stroke patients compensate by using higher-order motor control regions upon fatal loss of the principal motor cortical area. For long-term effects, rTMS for stroke treatment is becoming more and more promising as positive findings are being replicated. In the current special topic, authors report in stroke patients how consecutive rTMS session resulted in movement improvement (Di Lazzaro et al.) but also increased tactile detection (Fujimoto et al.). Sample size and gender effects need attention in stroke research as they seem to interact when using rTMS as a treatment tool (Chalah et al.; Di Lazzaro et al.). Last, patients with chronic pain not responsive to pharmacological treatment may benefit from NIBS tools over the primary motor region (DosSantos et al.), whereby distant changes in cortico-subcortical structures and neurotransmitter modulation (serotonin, GABA, glutamate) were associated to clinical improvement.

There is also a novel contribution from light therapy used as a NIBS protocol (see Johnstone et al.). The use of light stimulation has been tested on animal models of PD and AD using low-level near infrared light (NIr) therapy (Shaw et al., 2010; De Taboada et al., 2011), reported to lessen behavioral deficits in both animal models. It is noted that this procedure did not produce any beneficial effects in AD or PD (Johnstone et al.). Only a non-controlled and non-randomized clinical report showed some improvement in speech, some aspects of cognition and gait after NIr therapy in PD patients (Maloney et al., 2010), which needs to be replicated in a larger sample in a better controlled study. Thus, based on valid animal models, Nlr therapy warrants evaluation in larger samples in well-controlled studies, with other targets, and selection of intracranial or extracranial approaches based on the disease, to allow future clinical application.

New avenues of positive results are also obtained in attempts to improve cognitive functioning. AD patients showed improved working memory after tDCS and this was associated to changes in high-frequency bands (Marceglia et al.). However, the use of associated paradigms such as exercise (Morris et al., 2016) or cognitive rehabilitation (Cappon et al.) will boost the cognitive remediation and positive effects.

## Future work

There are a number of parallel issues across the therapeutic applications of NIBS that need to be addressed. The ultimate value of NIBS rests on proving it to be an efficient and long-lasting therapy that alleviates patient's specific psychiatric, neurological or cognitive symptomatology. However, the questions of *where, how* and *when* to stimulate are essential to be addressed in order to follow the logical steps to reach maximal NIBS efficacy for different symptoms and disorders. Although candidate cortical regions to act as targets for receiving NIBS are somewhat more clear for some neurological conditions, other neurological and psychiatric disorders still require evidence from imaging and physiological studies to identify the region or network to be targeted with NIBS. A critical factor is the inclusion of repeated stimulation sessions to achieve potentiation effects. This may be done with an initial period of daily stimulation for example 5 days of consecutive stimulation, followed by once a week booster sessions. Other procedural issues may also influence the quality and efficacy of the NIBS such as the state or subject dependency of the effects, use of neuronavigation vs. EEG localization of the target and these require due attention in future investigations. New methods to better quantify potential beneficial effects of NIBS are the use of models that account for long-term effects (Mahmud and Vassanelli). In future trials, to ensure that NIBS is cost-effective compared to standard medical therapy, there is a need to maximize the efficacy and positive outcomes of NIBS protocols. Sham-controlled randomized trials of NIBS are essential. Moreover, if the symptoms to be treated have a high within subject variability (e.g., pain, tinnitus and psychiatric symptoms) the clinical trials required could be even more complex and expensive. Using a telemedicine approach and/or using smartphone and wearable technology, continuous patient evaluation can be easier. This will allow NIBS technologies to be tested in a more efficient way.

It is also extremely important to reconsider NIBS variability at individual level. The same target with the same NIBS protocol may produce different effects in different individuals. A personalized approach is needed to reduce this source of variability. Nowadays, many technological tools are available for evaluating central nervous system disorders. However, the general approach is to apply a single therapy or an isolated technology to find the way to help a group of patients that have a common etiology but sometimes very different nervous system pathology and clinical presentations. It is necessary to find the perfect combination of assessment methods to evaluate symptoms and their change after application of a smart mix of therapeutic options, applied at a given time and at the appropriate “doses” to face the great complexity of neurological and psychiatric problems. This may be one of the future strategies for NIBS therapies to find a place in psychiatric and neurological clinics.

Finally, there is a need for safe, efficient and cost-effective NIBS methods such as transcranial static magnetic field stimulation (tSMS) or tDCS that can be portable and usable in patients' homes, which would facilitate generalization of the treatment to the patients' daily life environment.

## Author contributions

All authors listed, have made substantial, direct and intellectual contribution to the work, and approved it for publication.

### Conflict of interest statement

The authors declare that the research was conducted in the absence of any commercial or financial relationships that could be construed as a potential conflict of interest.
